# Linalool isomerase, a membrane-anchored enzyme in the anaerobic monoterpene degradation in *Thauera linaloolentis* 47Lol

**DOI:** 10.1186/s12858-016-0062-0

**Published:** 2016-03-15

**Authors:** Robert Marmulla, Barbara Šafarić, Stephanie Markert, Thomas Schweder, Jens Harder

**Affiliations:** Department of Microbiology, Max Planck Institute for Marine Microbiology, Celsiusstr. 1, D-28359 Bremen, Germany; Institute for Pharmacy, Department of Pharmaceutical Biotechnology, University of Greifswald, Felix-Hausdorff-Str. 3, D-17487 Greifswald, Germany

**Keywords:** Linalool, Geraniol, *Thauera*, Isomerase, Allyl alcohol, Monoterpene

## Abstract

**Background:**

*Thauera linaloolentis* 47Lol uses the tertiary monoterpene alcohol (*R,S*)-linalool as sole carbon and energy source under denitrifying conditions. The conversion of linalool to geraniol had been observed in carbon-excess cultures, suggesting the presence of a 3,1-hydroxyl-Δ^1^-Δ^2^-mutase (linalool isomerase) as responsible enzyme. To date, only a single enzyme catalyzing such a reaction is described: the linalool dehydratase/isomerase (Ldi) from *Castellaniella defragrans* 65Phen acting only on (*S*)-linalool.

**Results:**

The linalool isomerase activity was located in the inner membrane. It was enriched by subcellular fractionation and sucrose gradient centrifugation. MALDI-ToF MS analysis of the enriched protein identified the corresponding gene named *lis* that codes for the protein in the strain with the highest similarity to the Ldi. Linalool isomerase is predicted to have four transmembrane helices at the N-terminal domain and a cytosolic domain. Enzyme activity required a reductant for activation. A specific activity of 3.42 ± 0.28 nkat mg * protein^−1^ and a k_M_ value of 455 ± 124 μM were determined for the thermodynamically favored isomerization of geraniol to both linalool isomers at optimal conditions of pH 8 and 35 °C.

**Conclusion:**

The linalool isomerase from *T. linaloolentis* 47Lol represents a second member of the enzyme class 5.4.4.4, next to the linalool dehydratase/isomerase from *C. defragrans* 65Phen. Besides considerable amino acid sequence similarity both enzymes share common characteristics with respect to substrate affinity, pH and temperature optima, but differ in the dehydratase activity and the turnover of linalool isomers.

**Electronic supplementary material:**

The online version of this article (doi:10.1186/s12858-016-0062-0) contains supplementary material, which is available to authorized users.

## Background

**Fig. 1:**
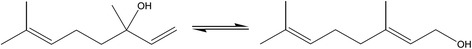
Linalool isomerase catalyzes the reversible reaction from linalool to geraniol

Monoterpenes (C_10_H_16_), naturally occurring hydrocarbons, are the main constituents of essential oils and belong to the diverse group of terpenoids, of which more than 50000 structures are known to date [1, 2]. They are produced as secondary plant metabolites and serve diverse functions like signaling, attraction/repellence of pollinators and insects, thermotolerance and are involved in allelopathy [3, 4]. Atmospheric monoterpene emission from plants was estimated to be 127 Tg C yr^−1^ [5], with half-lives of minutes to hours in the atmosphere due to their susceptibility to chemical and photooxidative reactions. Monoterpenes enter soils by precipitation from the atmosphere, excretion from roots and by leaf fall [6–9]. The hydrophobic character of monoterpenes causes cell toxicity, mainly by accumulation into and destabilization of the cell membranes [10]. Below toxic concentrations, microorganisms can use monoterpenes as carbon and energy source for growth. Several bacteria have been described to transform monoterpenes in the presence of oxygen as a cosubstrate applying mono- and dioxygenases [11], but also other biotransformations are described [12]. Linalool, a tertiary monoterpene alcohol (Fig. 1), is the main component of essential oils in lavender and coriander. Its chemical structure prevents a direct oxidation of the hydroxyl group. Hence, another functionalization or isomerization is the initial biological degradation reaction. Linalool is oxidized to 8-hydroxylinalool under aerobic conditions [13, 14]. In the absence of oxygen, the primary alcohol geraniol is formed in linalool-grown cultures of *Thauera linaloolentis* 47Lol (Fig. 1) [15]. This betaproteobacterium was isolated on linalool as sole carbon end energy source under denitrifying conditions [16]. A 3,1-hydroxyl-Δ^1^-Δ^2^-mutase was proposed as novel enzymatic function initializing the mineralization of linalool [15, 16].

A similar reaction was described for the bifunctional enzyme linalool dehydratase/isomerase from *Castellaniella defragrans* 65Phen. The enzyme catalyzes the reversible hydration of β-myrcene to (*S*)-linalool and its isomerization to geraniol. It is an oxygen-sensitive, periplasmatic protein of 43 kDa including a signal peptide for export [17, 18]. The tertiary alcohol 2-methyl-3-buten-2-ol (232-MB) may also be transformed by enzyme-catalyzed isomerization reactions. 232-MB is a metabolite in bacterial degradation of fuel oxygenates. Its mineralization may proceed via an initial isomerization to 3-methyl-2-buten-1-ol (prenol) in *Aquincola tertiaricarbonis* L108 and *Methylibium petroleiphilum* PM1 [19] as well as in *Pseudomonas putida* MB-1 [20]. The later was isolated on 232-MB as sole carbon source [20]. However, the corresponding enzymes were so far not characterized. For intramolecular hydroxyl-group transfer (EC 5.4.4.x), only seven different enzyme activities are described: (hydroxyamino) benzene mutase (EC 5.4.4.1), isochorismate synthase (EC 5.4.4.2), 3-(hydroxyamino) phenol mutase (EC 5.4.4.3), geraniol isomerase (EC 5.4.4.4), 9,12-octadecadienoate 8-hydroperoxide 8*R*-isomerase (EC 5.4.4.5), 9,12-octadecadienoate 8-hydroperoxide 8*S*-isomerase (EC 5.4.4.6) and hydroxyperoxy icosatetraenoate isomerase (EC 5.4.4.7).

We report the enrichment of the linalool isomerase activity in protein fractions of *Thauera linaloolentis* 47Lol and the kinetic properties of the enzyme. A corresponding gene was identified in the draft genome. We suggest to place the linalool isomerase of *Thauera linaloolentis* 47Lol as a new member in the enzyme family of intramolecular hydroxyl group transferases (EC 5.4.4.x) with the EC number 5.4.4.4 next to the geraniol isomerase function of the linalool dehydratase/isomerase from *Castellaniella defragrans* 65Phen.

## Results and discussion

### Identification of a candidate protein for linalool isomerase

The linalool dehydratase/isomerase (Ldi, NCBI:CBW30776) was used in similarity searches to identify a putative linalool isomerase protein in a draft genome of *T. linaloolentis* 47Lol. One protein showed a considerable similarity with an overall amino acid identity of 20 % (positives 33 %, E-value 3E-10, NCBI:ENO87364, Additional file 1: Figure S1). The corresponding gene is isolated from the adjacent genes (>150 bp) and encodes a protein of 644 amino acids with a calculated molecular weight of 71.8 kDa, an isoelectric point of 6.06 and a hydrophobicity of −0.115 (GRAVY) [21]. No signal peptide was predicted by the SignalP software. For the N-terminus, four transmembrane domains within the first 139 amino acids were predicted together with a localization in the inner membrane with the C-terminal protein fold in the cytoplasm. Conserved domains as described in Pfam were not present. The specific hydrophobicity values (GRAVY) were 0.94 and −0.406 for the N- and C-terminal parts of the protein (amino acids 1–139 and 140–644, respectively). The similarity to the Ldi was restricted to the C-terminal domain. Such a location at the cytoplasmic site of the inner membrane seems to be ideal for a catabolic enzyme acting on a hydrophobic substrate. This may maximize the contact with the substrate and reduces the intracellular concentration, but also produces geraniol for the next catabolic enzymes that likely depend on cytoplasmatic NAD^+^ as electron acceptor. In contrast, the periplasmatic location of Ldi is optimal for a defense enzyme. Myrcene is less toxic than the alcohols and can diffuse into the environment of the cell, thus keeping the damage at the inner membrane to a minimum.

### Enrichment of the linalool isomerase activity (Lis)

Lis activity was determined as geraniol isomerase activity and was detected in crude cell-free protein extracts also containing membrane fragments after application of high pressure cell disruption. Cell disintegration by osmosis or ultra sonification retained the activity in the membrane fraction. Due to the large cytoplasmatic domain of the candidate protein, we attempted the isolation as soluble enzyme from dialyzed crude extracts. The pH was increased to 9.5 to eventually increase the anionic character of the enzyme. The enzyme activity did not bind to a DEAE column. After ammonium sulfate addition (10 % v/v of a saturated solution), the enzyme activity was retained on a Phenyl-Sepharose column and eluted with pure water. A strong binding to hydrophobic columns has also been observed for Ldi [17] and other monoterpene-transforming enzymes [22]. A size determination of the eluted fraction on a size-exclusion column yielded a molecular weight of over 600 kDa, as native protein in phosphate buffer as well as in the presence of urea as denaturant. Visualization of the proteins on SDS-PAGE revealed the presence of many proteins, including abundant proteins between 60 and 70 kDa and around 33 kDa (data not shown).

**Fig. 2:**
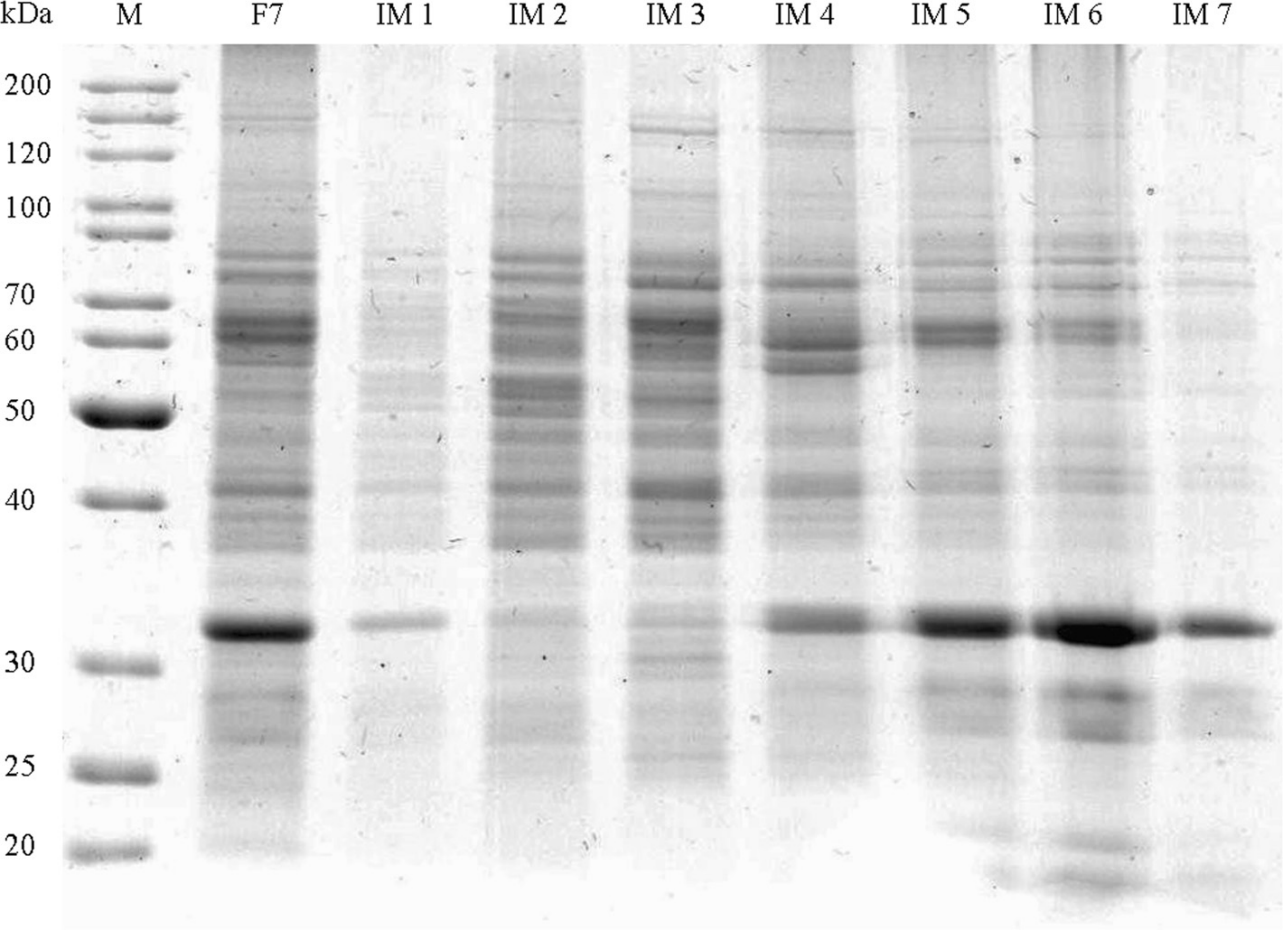
SDS-PAGE of sucrose gradient fractions after separation of the inner membrane (IM) fraction from spheroplast disintegration. (F7) inner membrane fraction from spheroplast disintegration, (IM 1) - (IM 7) fractions 1–7 (top-to-bottom) of sucrose gradient

**Fig. 3:**
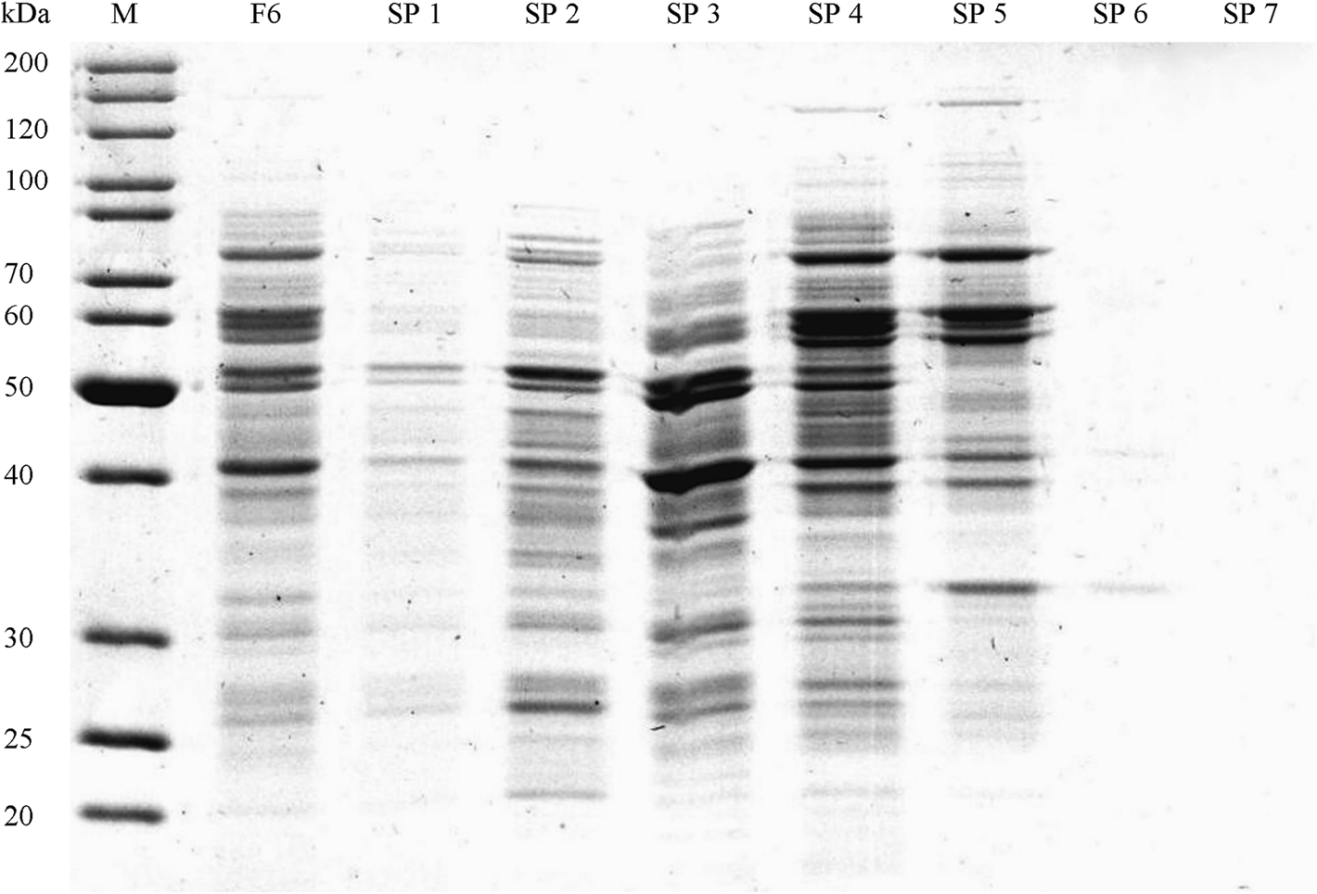
SDS-PAGE of sucrose gradient fractions after separation of the cytoplasmatic soluble protein fraction from spheroplast disintegration. (F6) Soluble protein fraction from spheroplast disintegration, (SP 1) - (SP 7) fractions 1–7 (top-to-bottom) of sucrose gradient

**Fig. 4:**
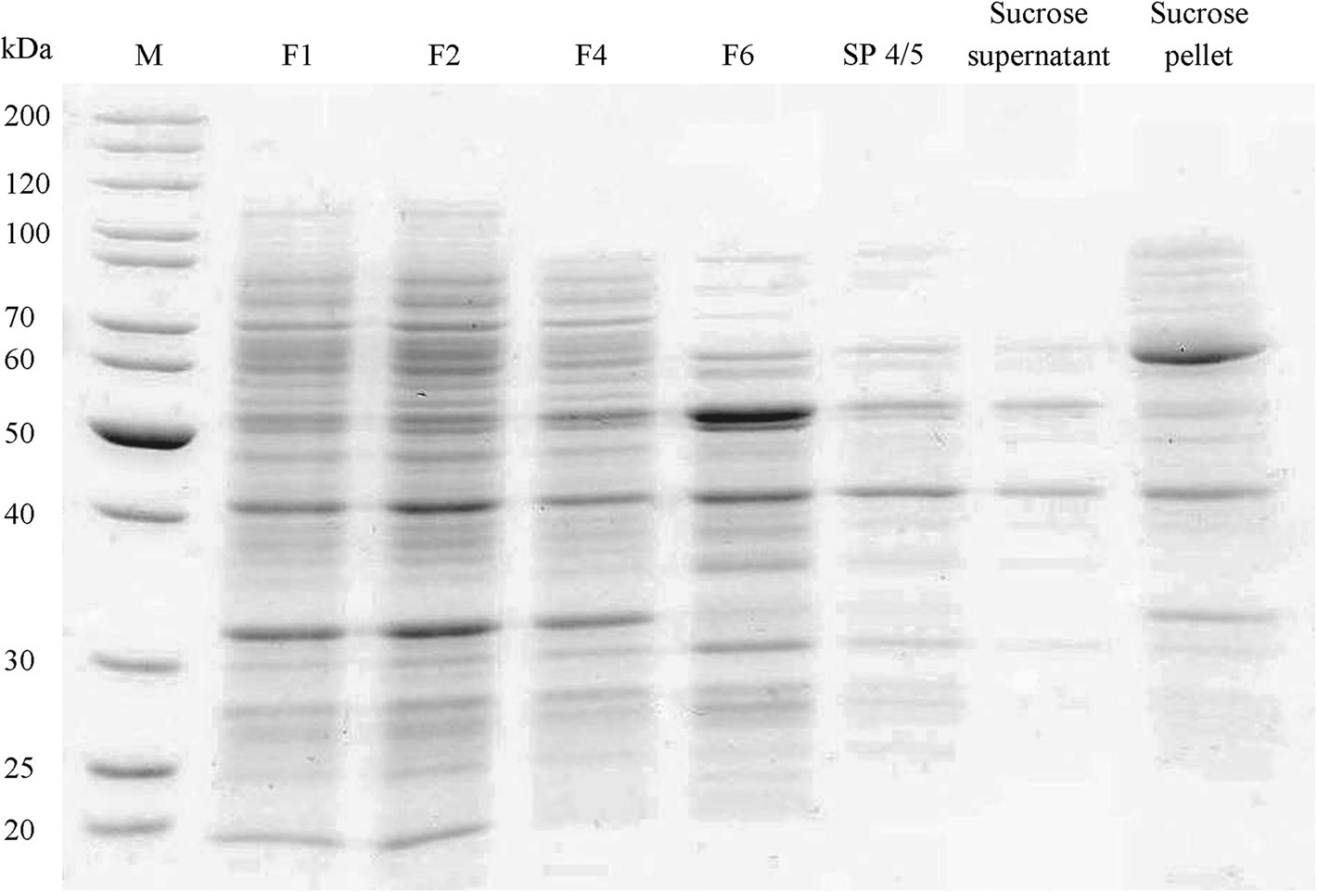
SDS-PAGE of Lis activity enrichment by spheroplast disintegration and sucrose gradient centrifugation. (F1) spheroplasts, (F2) spheroplasts after cell disintegration, (F4) crude extract after spheroplast disintegration, (F5) cytoplasmatic soluble protein fraction, (SP 4/5) fractions 4 and 5 from sucrose gradient centrifugation, (Sucrose supernatant) supernatant of second ultracentrifugation, (Sucrose pellet) protein pellet of second ultracentrifugation

The large size together with the predicted membrane association of the Lis candidate gene suggested an alternative purification approach, a subcellular fractionation including a sucrose-gradient centrifugation for the identification of membrane-associated proteins. First, the outer membrane and periplasmatic proteins were removed by spheroplast formation. After disintegration by high pressure release, the inner membrane (IM) fraction was separated from the cytosolic soluble protein (SP) fraction by ultracentrifugation (158 Svedberg). Total Lis activity was five times higher in the membrane fraction than in the soluble fraction (15.8 and 3.3 nkat, respectively) (Table 1 and Additional file 2: Figure S2). Both fractions were separated on sucrose gradients. The Lis activity of the membrane fraction (Fig. 2 and Table 2) was concentrated in two fractions with a sucrose density between 1.17 and 1.22 g mL^−1^ (IM4 and IM5). For the soluble protein fraction (Fig. 3 and Table 3), Lis activity was also concentrated in the fractions with the aforementioned sucrose content (SP4 and SP5). Protein gels revealed an enrichment of proteins with a molecular size between 60 and 70 kDa and around 32 kDa for the active fractions from IM and SP. Lis activity was deposited from the active fractions (SP4 and SP5) by decrease of the sucrose concentration and ultracentrifugation. SDS-PAGE characterized the dissolved pellet as containing an approx. 60 kDa protein as most abundant protein (Fig. 4 and Table 4). This purification stage was used for enzyme kinetics.

**Table 1:** Enrichment of Lis activity from spheroplasts of *T. linaloolentis* 47Lol

Sample	Protein [mg]	Activity [pkat]	Specific activity [pkat mg^−1^]	Relative specific activity	Protein yield [%]*
F1	71.9	7295	101		
F2	79.0	7800	99	1.0	100.0
F3	67.6	3060	45	0.5	
F4	51.8	24984	482	4.9	65.6
F5	29.1	513	18	0.2	36.8
F6	38.0	3283	86	0.9	73.3
F7	21.4	15848	741	7.5	41.3

(F1) spheroplasts, (F2) lysed spheroplasts, (F3) outer membrane and periplasmatic proteins, (F4) crude extract from spheroplasts, (F5) unbroken spheroplast and cell debris, (F6) cytoplasmatic, soluble proteins, (F7) inner membrane fraction. *F4 and F5 are derived from F2. F6 and F7 are derived from F4

**Table 2:** Enrichment of Lis activity by sucrose gradient centrifugation from inner membrane pellets (F7) obtained from spheroplasts of *T. linaloolentis* 47Lol. 1 mL fractions from top to bottom are shown

Sample	Protein [mg]	Activity [pkat]	Specific activity [pkat mg^−1^]	Relative specific activity	Protein yield [%]
Applied sample (F7)	6.74	5925	879	1	100.0
IM 1	0.20	139	697	0.8	3.0
IM 2	1.06	238	225	0.3	15.7
IM 3	1.44	870	604	0.7	21.4
IM 4	1.92	3085	1607	1.8	28.5
IM 5	3.28	3513	1071	1.2	48.7
IM 6	1.44	1050	729	0.8	21.4
IM 7	0.02	0	0	0.0	0.3

**Table 3:** Enrichment of Lis activity by sucrose gradient centrifugation from cytoplasmatic soluble protein fraction (F6) obtained from spheroplasts of *T. linaloolentis* 47Lol. 1 mL fractions from top to bottom are shown

Sample	Protein [mg]	Activity [pkat]	Specific activity [pkat mg^−1^]	Relative specific activity	Protein yield [%]
Applied sample (F6)	9.2	1863	202	1.0	100.0
SP 1	0.6	14	24	0.1	6.1
SP 2	1.5	16	11	0.1	16.7
SP 3	3.1	165	54	0.3	33.2
SP 4	2.7	273	100	0.5	29.5
SP 5	1.2	141	114	0.6	13.3
SP 6	0.3	13	47	0.2	3.0
SP 7	0.3	0	0	0.0	2.8

**Table 4:** Enrichment of Lis activity from spheroplasts of *T. linaloolentis* 47Lol by sucrose gradient centrifugation

Sample	Protein [mg]	Activity [pkat]	Specific activity [pkat mg^−1^]	Relative specific activity	Protein yield [%]
F1	40.8	1408	35		
F2	111.7	3130	28	1	100.0
F4	94.7	13572	143	5	84.8
F6	58.8	3440	59	2	52.6
SP 4/5	3.7	745	200	7	3.3
Sucrose supernatant	-	-	-	-	-
Sucrose pellet	1.9	323	171	6	1.7

(F1) spheroplasts, (F2) lysed spheroplasts, (F4) crude extract, (F6) cytoplasmatic, soluble protein fraction, (SP 4/5) fractions 4 and 5 from sucrose gradient, (Sucrose supernatant) supernatant after second ultracentrifugation, (Sucrose pellet) protein pellet after second ultracentrifugation

Particles with a Svedberg constant of above 158 S were separated from the soluble fraction. High pressure cell disintegration produces a number of small membrane fragments and vesicles. Small vesicles (microsomes) settle with a sedimentation coefficient between 100 and 10000 S [23]. We conclude from our observations that the Lis activity is located on a protein associated with the inner membrane. Only a minor fraction of the total enzyme activity, present as small membrane-protein aggregates, was enriched in the fraction that is traditionally considered to contain only cytosolic proteins.

The protein gels showed consistently protein(s) between 60 and 70 kDa in fractions with Lis activity. MALDI-ToF mass spectroscopy identified the protein band at approx. 60 kDa from several gels as NCBI:ENO87364, the protein that was predicted to be a candidate for the linalool isomerase. The discrepancy between the calculated molecular weight of 71.8 kDa and the observed weight of between 60 and 70 kDa is likely a result of the hydrophobic nature of the protein. Hydrophobic proteins, including membrane proteins tend to bind more SDS and show a faster migration in denaturing gel electrophoreses [24].

Further purification of the Lis activity was not successful, as a detergent-mediated release of the protein from the membrane was impaired by irreversible inhibitory effects on the enzyme activity. Detergents aid solubility of membrane proteins but also have an adverse effect on stability and functionality of proteins [25, 26]. Terpenoid synthases were shown to have a rather hydrophobic core shielding the catalytic center from surrounding bulk solvent [27]. Also the Lis may have a hydrophobic protein domain sensitive to detergents that change the conformation into a non-active state.

### Heterologous gene expression

**Fig. 5:**
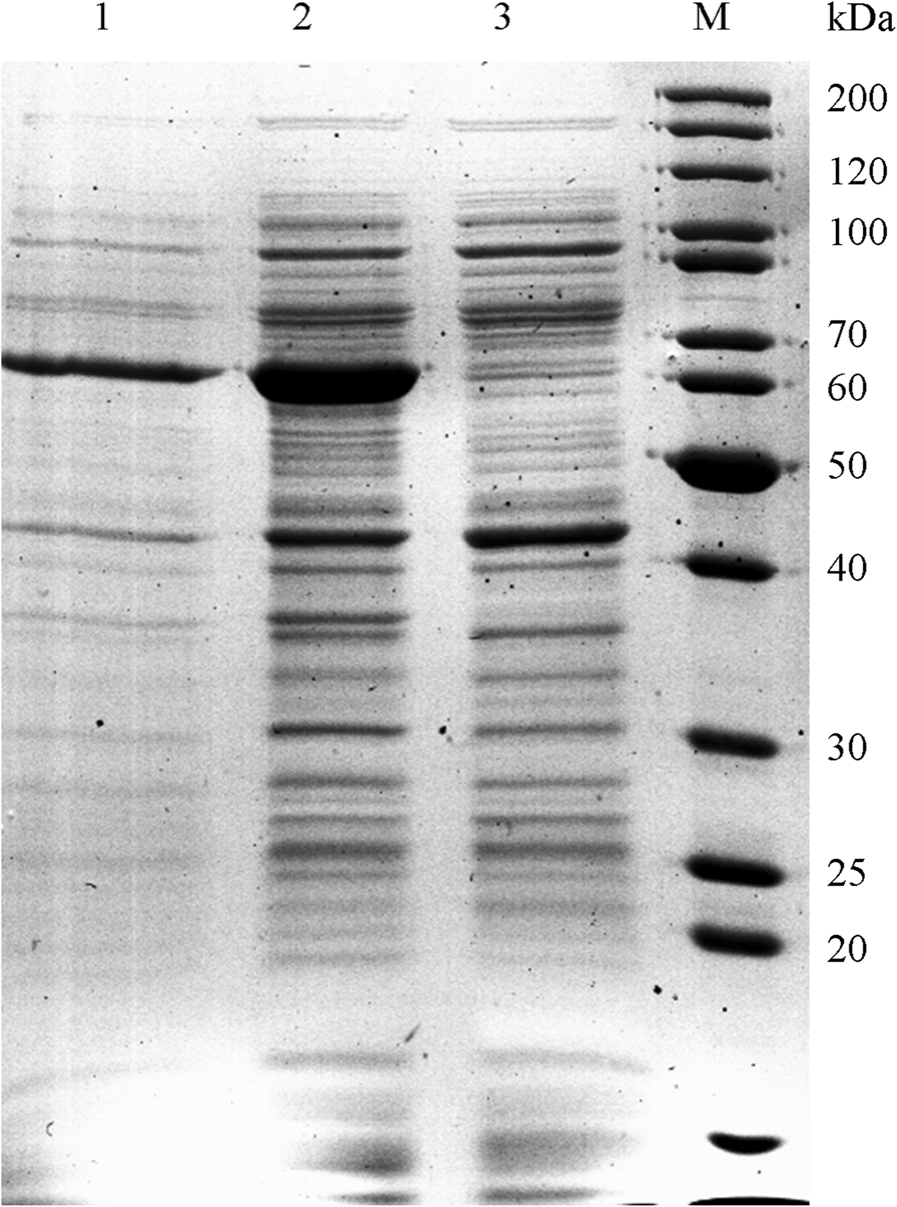
SDS-PAGE of heterologous expression of the *Lis* gene in *E. coli* BL21(DE3) pET42-Lis. (1) Cells from induced culture, (2) crude protein extract after cell disintegration and (3) soluble protein fraction after ultracentrifugation

Expression of the native *lis* gene in *E.coli* BL21(DE3) yielded an induced protein band around 60 kDa (Fig. 5). No inclusion bodies were observed. The expressed protein was located in the membrane fraction, but Lis activity was not observed in crude cell extracts or in cell-free protein fractions. Expression of a N-terminally truncated version of the linalool isomerase, representing the cytosolic part of the enzyme only, yielded a soluble protein that also did not show activity. The *lis* gene did not have rare codons. The folding in *E.coli* may have been incorrect.

### Characterization of linalool isomerase activity

**Fig. 6:**
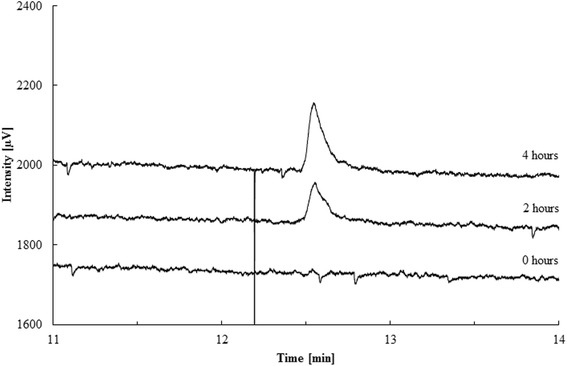
Formation of geraniol by linalool isomerase. The most enriched protein sample after sucrose gradient centrifugation and precipitation was used: 200 μL sample (1.04 mg mL^−1^ protein), 5 mM dithionite, 1 μL (*R,S*)-linalool (≈30 mM). Incubation was performed at 28 °C and individual samples were extracted with 200 μL n-hexane after 0, 2 and 4 h (from bottom to top) and measured by gas chromatography (Shimadzu GC 14A, see methods). Retention time of geraniol was at 12.55 min

**Fig. 7:**
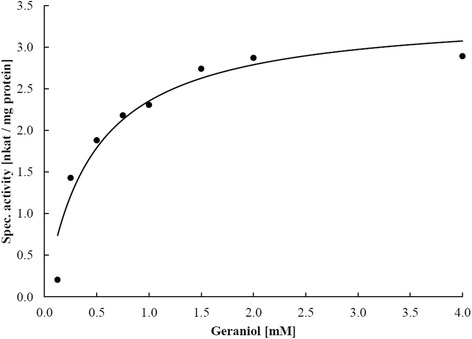
Michaelis-Menten plot of geraniol isomerization by linalool isomerase. An apparent k_M_-value and V_max_ value of 455 ± 124 μM and 3.42 ± 0.28 nkat * (mg protein)^−1^ were determined for the isomerization of geraniol to linalool

**Fig. 8:**
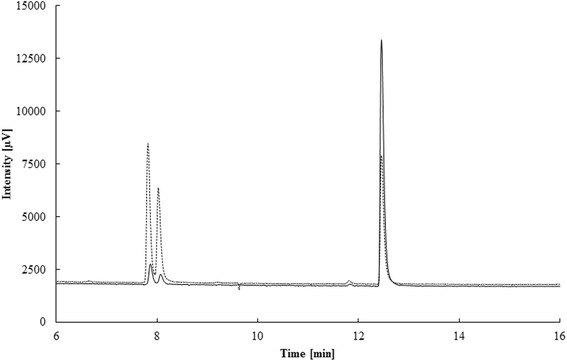
Gas chromatogram demonstration of the absence of stereoselectivity of the linalool isomerase. Soluble protein extract (1.4 mg protein; solid line) and inner membrane-enriched fraction (1.6 mg protein; dashed line) from spheroplast disintegration were incubated with geraniol, subsequently extracted with n-hexane and analyzed by gas chromatography. Retention times: (*R*)- and (*S*)-linalool 7.85 and 8.05 min, geraniol 12.45 min

The Lis activity was enriched without a chemical reductant in the buffer and enzymatically inactive. Activity was restored by addition of a reducing agent and under anoxic conditions in the enzyme assay. The activation could be initiated by cationic (ferrous iron), neutral (dithiothreitol) or anionic (dithionite) compounds. However, maximum activity was measured with 4 mM dithionite (268 pkat * mg protein^−1^). A large excess of reductant caused a decrease in activity, e.g. 10 mM dithionite or 8 mM DTT were less suitable for activation (Table 5). Lis activity was determined in the thermodynamically favorable direction from geraniol to linalool [15] and was observed between pH 7 and 9.5, with an optimum around pH 8. The temperature optimum was 35 °C and the activation energy was 80.4 ± 6.9 kJ mol^−1^. For comparison, the pH and temperature optimum of the Ldi enzyme were at pH 9 and 35 °C, respectively, and the activation energy was 68.6 kJ mol^−1^ [17]. Geraniol formation from linalool was detected within 4 h of incubation (Fig. 6). Kinetic parameters were determined with the most enriched sample in duplicates for the geraniol isomerization to linalool. The enzyme followed Michaelis-Menten-kinetics with an apparent k_M_-value of 455 ± 124 μM and a V_max_ of 3.42 ± 0.28 nkat * mg protein^−1^ (Fig. 7). A similar substrate affinity was determined for the linalool dehydratase/isomerase (k_M_ 500 μM). Maximal velocity was higher for the Ldi (V_max_ 410 nkat * mg protein^−1^) than for the Lis, however, the purification level for Lis was lower, and we do not know whether part of the enzyme is in an inactive state. Lis did not show a stereospecificity towards linalool isomers: both (*R*) and (*S*)-linalool were formed (Fig. 8). In contrast, the Ldi accepts only (*S*)-linalool as a substrate [17].

**Table 5:** Influence of different reducing agents on Lis activity

Reducing agent	Reduction potential [mV]	Concentration [mM]	Specific activity [pkat * mg protein^−1^]
Dithionite	- 660	2	89
		4	268
		10	210
Dithiothreitol	- 330	2	199
		8	145
		16	83
Cysteine	- 220	5	23
		10	7
Ferrous iron	- 236 (Fe(OH)_3_/Fe^2+^)	5	112

Earlier studies already showed the regiospecific formation of geraniol from linalool without nerol formation [15]. To confirm the regioselectivity, Lis activity was tested with nerol or citronellol and in combination with geraniol. No activity was measured with nerol or citronellol alone. Linalool isomerase activity dropped to approx. 50 % in the presence of nerol. Activity in the presence of citronellol and geraniol was barely detectable. Thus, the enzyme is regioselective and seems to bind nerol with a similar affinity as geraniol, whereas citronellol which lacks the C2-C3 double bond is stronger bound than geraniol.

UV-VIS spectroscopy in a range of 200–800 nm did not provide evidence for the presence of cofactors. Cofactor-independent enzymes are known for allylic rearrangements that are catalyzed by acid-base mechanisms [28]. The isomerization of geraniol to linalool requires a protonation of the hydroxyl group to leave as water, and a shift in electron density leading to a tertiary carbocation intermediate which may be attacked by water or a hydroxyl ion, resulting in the formation of linalool.

## Conclusion

We identified a linalool isomerase in *T. linaloolentis* 47Lol by partial protein purification. The gene encodes a two-domain protein with a N-terminal anchor in the inner membrane that is characterized by four transmembrane helices and a C-terminal cytosolic domain which showed considerable similarity to the linalool dehydratase/isomerase from *C. defragrans* 65Phen. The enzyme is active in the reduced state and sensitive towards detergents. It expands the enzyme class of intramolecular hydroxyl group transferases as a new member, linalool isomerase (5.4.4.4).

## Methods

### Bacterial strains, cultivation conditions and biomass harvest

*T. linaloolentis* 47Lol was cultivated under anaerobic, denitrifying conditions in artificial fresh water (AFW) medium. Medium was prepared as described by Foss et al. [29] with modifications: carbonate buffer was replaced by 10 mM Na_2_HPO_4_/NaH_2_PO_4_ and vitamins were omitted. The headspace contained only nitrogen gas. 1–2 mM (*R,S*)-linalool (>97 % purity; Sigma-Aldrich, Germany) were directly applied without carrier phase as sole carbon and energy source. Cultures were incubated at 28 °C under mild shaking (60 rpm). Alternatively, they were stirred. Bacterial biomass for protein purification was obtained from 2 L cultures grown on 2 mM linalool and 20 mM nitrate by centrifugation (16000 × *g*, 25 min, 4 °C). If not used directly, biomass was frozen in liquid nitrogen and stored at −80 °C.

### Purification attempts by chromatography

First attempts on the purification of the linalool isomerase were performed by classical column chromatography using anion exchange and hydrophobic interaction columns (DEAE; binding buffer: 80 mM Tris-Cl, pH 9.5, elution buffer: 80 mM Tris-Cl, pH 9.5 with 1 M NaCl; Phenyl Sepharose; binding buffer: 80 mM Tris-Cl, pH 8.0 with 10 % v/v of a saturated ammonium sulfate solution, elution buffers: 80 mM Tris-Cl, pH 8.0 and water). Size-exclusion chromatography was performed on a HiLoad 16/60 Superdex200 column (GE healthcare, dimensions: 16 × 600 mm; 20 mM KH_2_PO_4_/K_2_HPO_4_, pH 8.0 with or without 6 M urea). Calibration was performed with thyroglobulin (670 kDa), bovine gamma-globulin (158 kDa), chicken ovalalbumin (44 kDa), equine myoglobin (17 kDa) and vitamin B12 (1.35 kDa).

Soluble protein extract was prepared by resuspending biomass in Tris-Cl buffer (80 mM, pH 9.5 for DEAE and pH 8.0 for HIC) and fast thawing at room temperature. Cell disintegration was performed by mechanical sheering using a One-Shot cell disruptor (Constant Systems Ltd., Daventry, UK) at 1.7 GPa two times. The crude extract was clarified by ultracentrifugation (150000 × *g*, 30 min, 4 °C).

All purification steps were performed on ice or at 5 °C. SDS-PAGE was used to characterize purification samples and protein concentrations were determined according to the method described by Bradford using bovine serum albumin as a calibration standard [30].

### Spheroplast preparation

Spheroplasts were prepared according to the protocol for subcellular fractionation described by Koßmehl et al. [31]. Cells were washed with 1.5 M NaCl solution, collected by centrifugation (14200 × *g*, 20 min, 4 °C) and resuspended in 20 % (w/v) sucrose, 30 mM Tris-Cl (pH 8.0) and 2 mM EDTA for osmotic shock treatment. The cell suspension was incubated for 20 min at 30 °C. A spheroplast enriched pellet was formed by centrifugation (14200 × *g*, 20 min, 4 °C). The pellet was resuspended in 5 mL of ice-cold 40 mM Tris-Cl (pH 8.0) and disintegrated by a One Shot Cell Disruptor (Constants Systems Ltd., UK) in two passages at 1.7 GPa. After removal of larger cell debris and unbroken cells by centrifugation (14200 × *g*, 20 min), the supernatant was further clarified by ultracentrifugation (104000 × *g*, 1 h, 4 °C). The resulting pellet and supernatant corresponded to an inner membrane fraction (IM, F7, Additional file 1: Figure S1) and a soluble protein fraction (SP, F6, Additional file 1: Figure S1), respectively.

### Sucrose density gradient centrifugation

A linear sucrose gradient was created by overlaying a 20 % (w/v) over a 70 % (w/v) sucrose solution, 3 mL each. The tubes were incubated horizontally for 90 min to allow mixing. A 1 mL-protein sample was loaded carefully on top of the gradient and centrifugation was performed in a L-70 ultracentrifuge (70.1Ti rotor, Beckmann Coulter) at 260000 × *g*, for 4 h at 4 °C, with slow acceleration and deceleration. The linearity of the sucrose gradient was confirmed by gravimetrical measurement. 1 mL fractions covering the gradient were analyzed for enzyme activity and for protein content on SDS-PAGE. The two most active fractions were pooled, diluted with 40 mM Tris-Cl (pH 8.0) to a final volume of 8 mL and a second ultracentrifugation step (260000 × *g*, 4 °C, 1.5 h ≡ 42 Svedberg) was performed to remove sucrose. This resulted in the formation of a protein pellet enriched in Lis activity, which was resuspended in 1 mL buffer and used for the characterization of the enzyme kinetic.

### Proteomics by MALDI-ToF MS

Protein samples, obtained from individual purifications, were analyzed by SDS-PAGE coupled with matrix-assisted laser desorption/ionization time of flight (MALDI-ToF) mass spectrometry (MS). Protein bands in gels were excised manually, and the Ettan Spot Handling Workstation (GE Healthcare) was used for trypsin digestion and embedding of the resulting peptide solutions in an α-cyano-4-hydroxycinnamic acid matrix for spotting onto MALDI targets. MALDI-ToF MS analysis was performed on an AB SCIEX TOF/TOF™ 5800 Analyzer (AB Sciex/MDS Analytical Technologies [32]. Spectra in a mass range from 900 to 3700 Da (focus 1700 Da) were recorded and analyzed by GPS Explorer™ Software Version 3.6 (build 332, Applied Biosystems) and the Mascot search engine version 2.4.0 (Matrix Science Ltd, London, UK) using the RAST draft genome as reference.

### Heterologous gene expression

The predicted *Lis* gene was isolated from genomic DNA of *T. linaloolentis* 47Lol by means of PCR, using the Phusion Polymerase according to the manufacturer manual (Life Technologies, Thermo Fisher Scientific, Waltham, USA) with the primer pair pLI_NdeI_FW (TCGTACATATGATGAGCAATATGGAATCG) and pLI_BglII_RV (CGATAGATCTTCAGTGGCCCGGCTTG, annealing temperature 59 °C). Additionally, a N-terminal truncated version of the gene was constructed with the primer pair pLI_NdeI_FW-truncated-N (TCGTACATATGATGCGCGGCGCCAAGC) and pLI_BglII_RV (annealing temperature 68 °C), which had an artificial start codon (ATG) and covered the amino acids 141–644 of the Lis. The genes were cloned into the pET42a overexpression plasmid and transformed into *E. coli* BL21(DE3). *E. coli* BL21(DE3) pET42-Lis or pET42-Lis-ΔN were grown in liquid LB medium at 37 °C and protein expression was induced by addition of 1 mM IPTG at an optical density (600 nm) of around 1. Cultures were further incubated at 30 °C for 4–5 h. Biomass was harvested by centrifugation (16000 × *g*, 25 min, 4 °C). Protein extracts were prepared as described above.

### Geraniol isomerase activity

Lis activity was determined by end-point analysis for the thermodynamically favored reaction from geraniol to linlaool. Subfractions obtained during purification were dialyzed and adjusted to Tris-Cl buffer (40 mM, pH 8.0). Individual assays were prepared in 4 mL glass vials in an anaerobic chamber with N_2_ headspace, containing 300–500 μL of sample and 5 mM dithionite as reducing agent. Samples were incubated for 20 min prior addition of 200 μL organic phase (200 mM geraniol in 2,2,4,4,6,8,8-heptamethylnonane, HMN). Vials were air-tight closed with butyl rubber stoppers and incubated for 14–16 h at 28 °C under mild shaking. Product formation was determined by gas chromatography with flame ionization detection (PerkinElmer Auto System XL, Überlingen, Germany). 1 μL of the HMN phase was injected onto an Optima-5 column (30 m × 0.32 mm, 0.25 μm film thickness; Macherey-Nagel, Germany) with hydrogen as carrier gas and the following temperature program: injection port 250 °C, detection port 350 °C, initial column temperature 40 °C for 2 min, increasing to 100 °C at a rate of 4 °C min^−1^, keeping 100 °C for 0.1 min, followed by an increase to 320 °C at 45 °C min^−1^ and hold for 3 min. The split ratio was set to 1:9.

The effect of detergents on Lis activity was tested for Triton X100 and Tween20 (0.5 %, 1 % w/v), CHAPS (0.1 % w/v) and n-octyl-α-D-glucoside (0.1 %, 0.5 %, 1 % w/v). Detergents were added to the soluble spheroplast fraction (protein concentration 0.1 mg mL^−1^) and aforementioned enzyme assays were performed.

Reducing agents were tested with a dialyzed (Visking dialysis tubing 12–14 kDa cut-off, Serva) soluble protein extract (4 mg mL^−1^ protein). The following reducing agents were added prior to the start of the assay: dithionite (2, 4 and 10 mM), dithiothreitol (2, 8 and 16 mM), cysteine (5 and 10 mM), or ferrous iron (5 mM).

Temperature dependency on Lis activity was determined between 12 and 50 °C. Samples (300 μL, 6.8 mg mL^−1^ protein) were pre-incubated for 20 min at the individual temperatures prior to substrate addition. The assay was terminated after 8 h and analyzed by gas chromatography. Activation energy was calculated from the Arrhenius plot (y = −9664.3 x + 36.2; R^2^ = 0.914).

The pH-optimum was determined by incubating crude cell lysate (20 mg mL^−1^) in a pH-range from 7 to 9.5 in Tris-Cl (40 mM) applying the aforementioned enzyme assay.

Kinetic parameters (k_M_ and V_max_) were determined for the most enriched enzyme fraction in biological duplicates (68 and 80 μg mL^−1^ protein; 10 to 20 μg total protein in final assay). Samples were incubated with geraniol concentration from 0.125 to 4 mM, directly applied without carrier phase. Both substrate and enzyme were prepared separately with 7 mM dithionite and pre-incubated. Reactions were started by injecting an equal volume (200 μL) of enzyme to the substrate solution. Samples were incubated at 28 °C for 90 min and terminated by addition of 100 mM NaOH (final concentration). 1 μL of sample was directly subjected to GC analysis. Kinetic parameters were calculated from primary data plotted in a Michaelis-Menthen-graph.

Substrate specificity of the Lis was tested with geraniol, nerol and citronellol. A 400 μL-sample (active fraction after sucrose gradient, SP 4/5) was incubated with 200 μL of 200 mM geraniol, nerol and citronellol in HMN as well as with 200 μL of geraniol-nerol and geraniol-citronellol mixtures in HMN (100 mM each). Assays were prepared as aforementioned.

Stereoselectivity was tested with soluble protein extract (1.4 mg protein) and inner membrane-enriched fraction (1.6 mg protein) from spheroplast disintegration. Samples were treated with 5 mM dithionite and incubated with 10 mM geraniol under anaerobic conditions at 28 °C for 14 h and subsequently extracted with 200 μL n-hexane. Monoterpene analysis was performed by gas chromatography with flame ionization detector (Shimadzu GC-14A, Shimadzu Corporation) on a Hydrodex-ß-6TBDM column (25 m × 0.25 mm, Macherey-Nagel, Germany) with the following temperature program: injection port 200 °C, detection port 250 °C, initial column temperature 60 °C for 1 min, increasing to 130 °C at a rate of 5 °C min^−1^, keeping 130 °C for 0.5 min, followed by an increase to 230 °C at 20 °C min^−1^ and hold for 4 min.

### Linalool isomerase activity

The forward reaction of the linalool isomerase - linalool to geraniol - was tested in a separate assay. 200 μL of enriched fraction after sucrose gradient centrifugation were treated with 5 mM dithionite and incubated with 1 μL (*R,S*)-linalool under anaerobic conditions at 28 °C for 0, 2 and 4 h. Samples were extracted with 200 μL n-hexane and analyzed by GC (Shimadzu GC-14A).

### UV-VIS spectrum for cofactors

The most enriched, active protein sample (0.95 mg mL^−1^ protein) was analyzed by UV-VIS spectroscopy (Beckman DU-640 spectrophotometer) in the range of 200–800 nm to detect cofactors.

### Gene identification and bioinformatic analysis

A draft genome for *T. linaloolentis* 47Lol was obtained by merging data from two at NCBI public available draft genomes: ASM31020 (4.199 Mbp on 220 contigs, published 2012) and ASM62130 (4.214 Mbp on 46 contigs, published 2014). Contigs were automatically merged using Sequencher 4.6 with a minimum match percentage of 95 % and a minimum overlap of 50 bp. The resulting draft genome had 4.4 Mbp on 23 contigs and was uploaded to RAST for further analysis [33, 34]. Identification of a putative gene, coding for a linalool isomerase (*Lis*), was performed by homology search using the linalool dehydratase/isomerase sequence from *Castellaniella degragrans* 65Phen. The identified gene was analyzed by various bioinformatic tools: SignalP 4.1 for prediction of signal peptides [35], TMHMM, SOSUI and Philius for prediction of transmembrane helices [36–39] and the Pfam database to search for motifs and domain patterns [40].

## Availability of data and materials

The data sets supporting the results of this article are included within the article and its additional files.

## Additional files

Additional file 1: Figure S1. Alignment of the linalool dehydratase/isomerase (NCBI:CBW30776) from *C. defragrans* 65Phen and the linalool isomerase from T. *linaloolentis* 47Lol (NCBI:ENO87364). Color indicates similarity. (PNG 106 kb) Additional file 2: Figure S2. SDS-PAGE of different fractions during the inner membrane preparation from spheroplasts. (F1) spheroplasts, (F2) spheroplasts after cell disintegration, (F3) outer membrane and periplasmatic protein fraction, (F4) crude extract after spheroplast disintegration, (F5) unbroken spheroplast and cell debris, (F6) cytoplasmatic, soluble protein fraction, (F7) inner membrane fraction. (PNG 539 kb)
