# Supplementary figures and images for: Linalool isomerase, a membrane-anchored enzyme in the anaerobic monoterpene degradation in Thauera linaloolentis 47Lol
Source: BMC Biochem. 2016 Mar 15;17:6. doi: 10.1186/s12858-016-0062-0 (PMC4791888; doi:10.1186/s12858-016-0062-0)

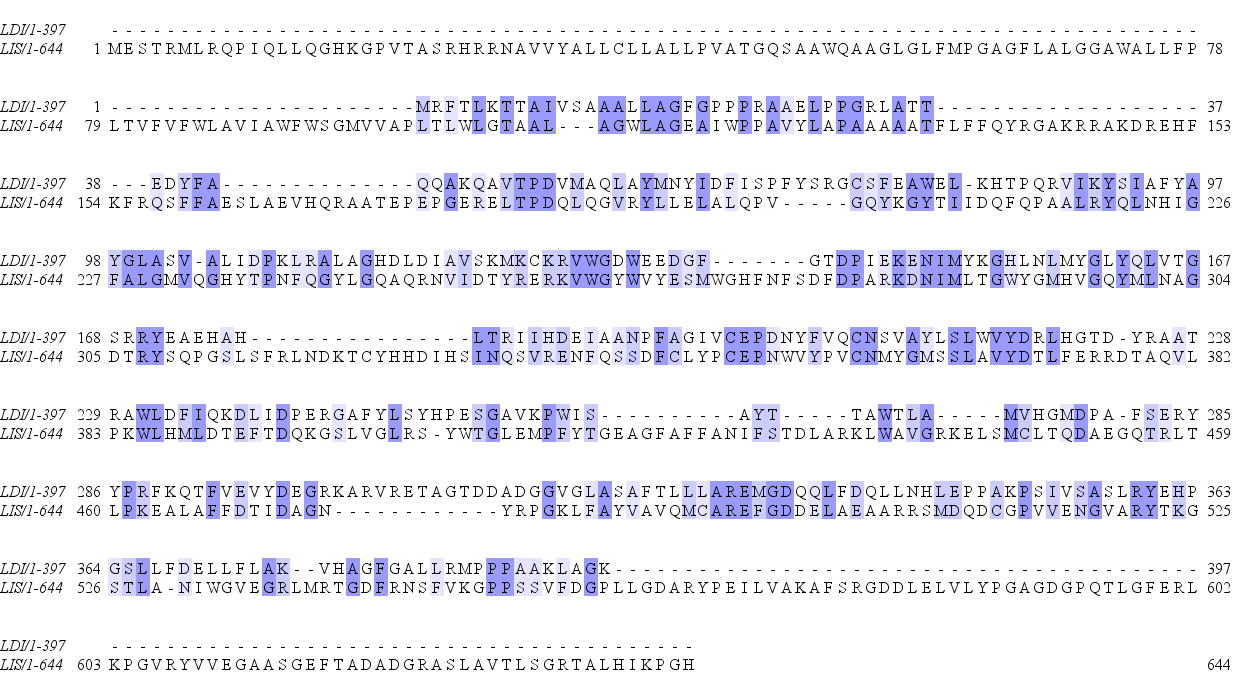

Supplement: Additional file 1: Figure S1. — Alignment of the linalool dehydratase/isomerase (NCBI:CBW30776) from C. defragrans 65Phen and the linalool isomerase from T. linaloolentis 47Lol (NCBI:ENO87364). Color indicates similarity. (PNG 106 kb) [file 12858_2016_62_MOESM1_ESM.png]

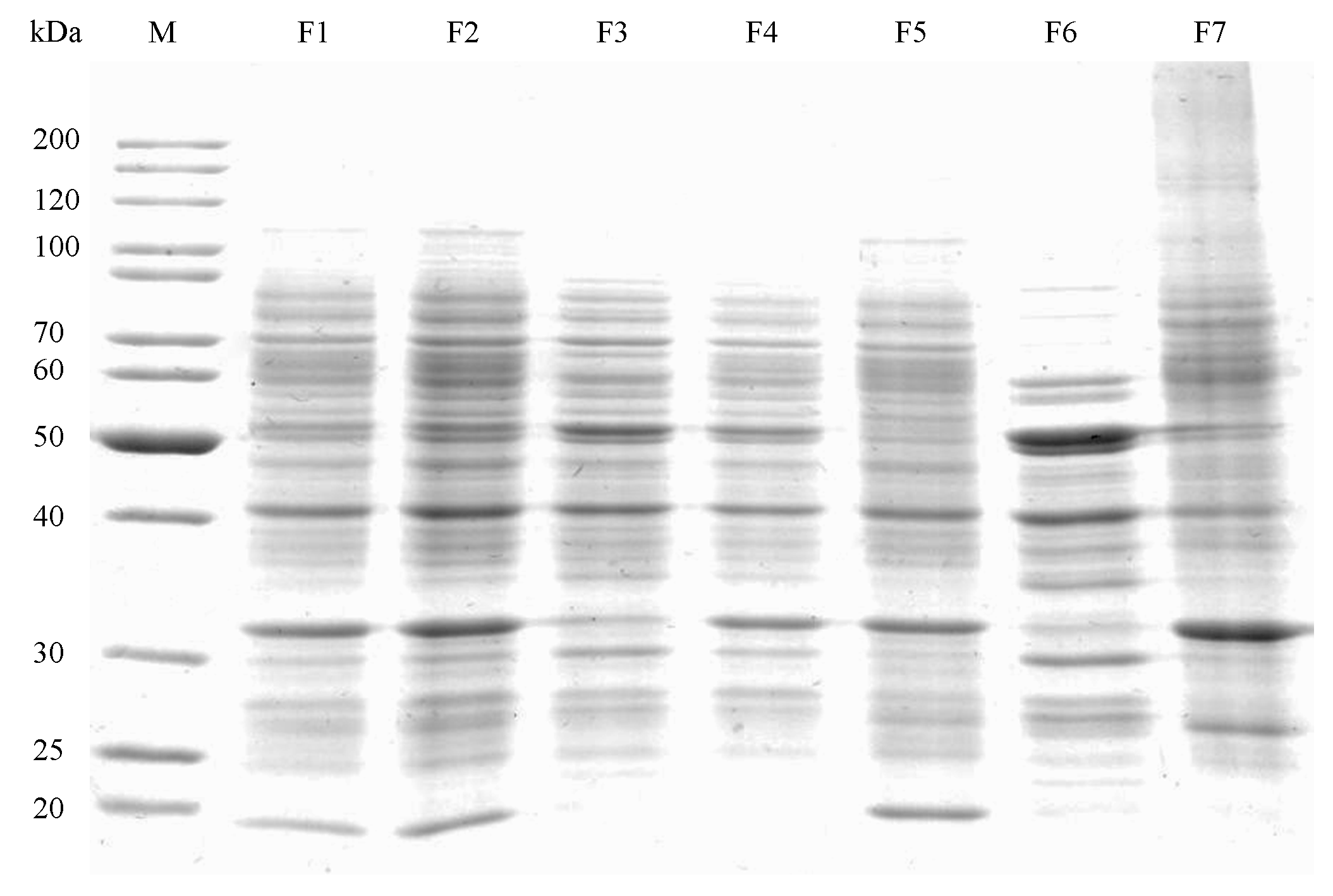

Supplement: Additional file 2: Figure S2. — SDS-PAGE of different fractions during the inner membrane preparation from spheroplasts. (F1) spheroplasts, (F2) spheroplasts after cell disintegration, (F3) outer membrane and periplasmatic protein fraction, (F4) crude extract after spheroplast disintegration, (F5) unbroken spheroplast and cell debris, (F6) cytoplasmatic, soluble protein fraction, (F7) inner membrane fraction. (PNG 539 kb) [file 12858_2016_62_MOESM2_ESM.png]
